# Social Connection in Times of Physical Distancing

**DOI:** 10.1093/geroni/igab046.132

**Published:** 2021-12-17

**Authors:** Jeongeun Lee

## Abstract

COVID-19 has been especially devastating to older adults. To prevent the spread of the virus, physical distancing has become the norm. As a result, there are fewer opportunities available for face-to-face interaction and social activities, which may be particularly harmful to older adults, given their existing loneliness levels. Thus, this symposium brings together a collection of papers that exemplify the interplay of social connection, activities, and mental health outcomes among older adults facing loneliness. The first paper will discuss how activity diversity is linked to higher loneliness and depressive symptoms among heterosexual and LGBTQ older adults. The second paper will present findings on the changes in social connectedness due to physical distancing and their associated impact on the mental health outcomes among older adults. The third paper will present qualitative findings on the effect of physical distancing on older adults' social connectedness using a mixed-method study. The final paper discusses the challenges faced by older adults in their use of digital media for social connection in the present pandemic and highlights some of the population's untapped strengths, which can be leveraged to help them live prosperous online lives. All papers will address practical tips and recommendations for actions, which key stakeholders can take to support older adults during the pandemic. The discussant, Dr. Kahana, will integrate the four papers and highlight the potential and limits of the current effort to address these issues and consider future inquiry routes.

